# Honeycomb-Structured Porous Films from Poly(3-hydroxybutyrate) and Poly(3-hydroxybutyrate-*co*-3-hydroxyvalerate): Physicochemical Characterization and Mesenchymal Stem Cells Behavior

**DOI:** 10.3390/polym14132671

**Published:** 2022-06-30

**Authors:** Viktoryia I. Kulikouskaya, Viktoryia V. Nikalaichuk, Anton P. Bonartsev, Elizaveta A. Akoulina, Nikita V. Belishev, Irina V. Demianova, Dariana V. Chesnokova, Tatiana K. Makhina, Garina A. Bonartseva, Konstantin V. Shaitan, Kseniya S. Hileuskaya, Vera V. Voinova

**Affiliations:** 1 Institute of Chemistry of New Materials, National Academy of Sciences of Belarus, 36 Skariny Str., 220141 Minsk, Belarus; kulikouskaya@gmail.com (V.I.K.); vica10bcn@gmail.com (V.V.N.); k_hilevskay@mail.ru (K.S.H.); 2 Faculty of Biology, M.V. Lomonosov Moscow State University, Leninskie Gory 1-12, 119234 Moscow, Russia; ant_bonar@mail.ru (A.P.B.); akoulinaliza@gmail.com (E.A.A.); nbelishev@gmail.com (N.V.B.); irinydem@yandex.com (I.V.D.); daryana8@yandex.ru (D.V.C.); shaytan49@yandex.ru (K.V.S.); 3 Biological Faculty, Shenzhen MSU-BIT University, Ruyi Rd. 299, Longgang District, Shenzhen 518172, China; 4 Federal Research Centre “Fundamentals of Biotechnology” of the Russian Academy of Science, Leninsky Prospect, 33, Build. 2, 119071 Moscow, Russia; tat.makhina@gmail.com (T.K.M.); bonar@inbi.ras.ru (G.A.B.)

**Keywords:** poly(3-hydroxybutyrate), poly(3-hydroxybutyrate-*co*-hygroxyvalerate), surface morphology, honeycomb, spin-coating, pores morphology, structure topography

## Abstract

Surface morphology affects cell attachment and proliferation. In this research, different films made of biodegradable polymers, poly(3-hydroxybutyrate) (PHB) and poly(3-hydroxybutyrate-*co*-3-hydroxyvalerate) (PHB-*co*-HV), containing different molecular weights, with microstructured surfaces were investigated. Two methods were used to obtain patterned films—water-assisted self-assembly (“breath figure”) and spin-coating techniques. The water-assisted technique made it possible to obtain porous films with a self-assembled pore structure, which is dependent on the monomer composition of a polymer along with its molecular weight and the technique parameters (distance from the nozzle, volume, and polymer concentration in working solution). Their pore morphologies were evaluated and their hydrophobicity was examined. Mesenchymal stem cells (MSCs) isolated from bone marrow were cultivated on a porous film surface. MSCs’ attachment differed markedly depending on surface morphology. On strip-formed stamp films, MSCs elongated along the structure, however, they interacted with a larger area of film surface. The honeycomb films and column type films did not set the direction of extrusion, but cell flattening depended on structure topography. Thus, stem cells can “feel” the various surface morphologies of self-assembled honeycomb films and change their behavior depending on it.

## 1. Introduction

Nowadays, functional biomaterial design with tailored characteristics is one of the most intensively progressing fields in the life sciences [1,2]. Such materials are of a great interest for tissue engineering and drug delivery applications, for cell-based biosensor technologies and microfluidic cell screening systems, etc. It is well known that biomaterial surface topography (size, geometric arrangement, and shape of surface features) can affect cell adhesion and allow the manipulation of the cellular function and behavior [1,2]. The nano and microstructure of biomaterials (primarily polymers) that form various surfaces of mammalian organs (mucosa, internal structures of bone and cartilage tissues, vessel endothelium, external surfaces of vessels and nerves) play a great role in the cell behavior regulation. Moreover, microscale topography can promote or restrict cell alignment, spreading, morphology and migration. Therefore, there is an intensifying interest in the use of micro-patterned biomaterials to control cell adhesion and growth on their surfaces [3]. Several studies [2,4,5,6,7] have shown that manipulation of the pore size in submicro- and micro-range allow to control attachment, growth and proliferation of cells (stem cells, fibroblasts, hepatocytes, etc.). For instance, Kawano and co-workers have discovered [8] that honeycomb-shaped surface microtopography could induce differentiation of human mesenchymal stem cells into osteospecific and myospecific directions depending on the pore and frame size. Numerous studies have shown the possibility of controlling the stem cell phenotype by using honeycomb-patterned films [5,7,9]. Honeycomb patterns also revealed a strong influence on hepatocyte adhesion, actin organization and expression of liver-specific functions [10]. Beattie and co-workers demonstrated [11] that patterned films with small pores (<1 μm) can promote the attachment and proliferation of fibroblasts when compared to films containing larger pores (>1 μm). Nishikawa and co-workers revealed that the shape of the pores can guide cardiac myocytes’ spreading. Cardiomyocytes were aligned along the long axis of the micropores with the shape of elongated hexagons and rectangles, forming fibrous tissue [12]. It has been confirmed in studies in vivo that the selected roughness of implants’ surfaces can regulate cellular behavior and enhance osseointegration [13], which gives potential for regulation of cellular differentiation without additional substances. 

Many approaches have been employed to create polymeric substrates with various surface geometric features. For instance, micro-patterned surfaces can be fabricated by lithography, microcontact printing or template techniques [14,15]. However, these techniques are rather complex, involve several steps and require specific and expensive equipment, along with high energy consumption [6]. In this regard, the fabrication of micro-patterned polymer films via breath figure technique appears to be really promising. This approach was first proposed by B. Francois to prepare honeycomb-patterned films from star-shaped polystyrene [16,17]. Micropatterned films can be obtained by evaporating the solvent of the cast polymer solution in a humid atmosphere [16,17,18,19]. The key steps of this process are the water microdroplets’ condensation onto the cold surfaces of the evaporating polymer solution and the water microspheres’ self-organization into a hexagonal array on the surface of the polymer solution due to Benard–Marangoni convection [18,19]. Porous honeycomb-like films with hexagonally arranged pores are produced after complete evaporation of the solvent. Compared to other methods, the breath figure technique has numerous advantages, which makes it one of the most widely used approaches to fabricate porous polymer films. This is a simple and low-cost technique that does not require specialized or expensive equipment, nor does it require multiple steps; it is fast and can produce highly ordered porous films from a variety of polymers. Micropores with different sizes and shapes can be easily tailored by controlling the process parameters (e.g., rate of air flow, humidity, polymer concentration, etc.).

Moreover, the water microdroplet technique is a more nature-oriented method because of the self-organization of the obtained polymer surface structures in contrast to the artificial rigid topography of polymer films obtained by other methods, for example, polymer melt-casting on lithography-patterned molds. This self-organized polymer topography can depend on the polymer physicochemical properties [20]. It is a great tool for creating new geometric elements that can affect the growth and differentiation of cells grown on them when it is possible to control the formation of such surface structures. This fully applies to the natural polymer, poly(3-hydroxybutyrate) (PHB), which is the progenitor of a whole class of biodegradable and biocompatible polymers—polyhydroxyalkanoates (PHAs). They are widely used in medicine for the manufacture of implantable medical devices, as well as for the preparation of scaffolds for tissue engineering [21]. Being partially crystalline, this biopolymer is able to form various microstructures on its surfaces upon the manufacture of different devices. It was shown that the microstructured surface of PHB films has an effect on cell proliferation and differentiation grown on them [10]. The use of a copolymer of PHB with 3-hydroxyvalerate will make it possible to study the influence of polymer characteristics (e.g., hydrophobicity and polymer chain structure) on the formation of the surface microstructure.

In this paper, the fabrication of honeycomb-patterned films from PHB and its copolymer with 3-hydroxyvalerate (poly(3-hydroxybutyrate-*co*-3-hydroxyvalerate, PHB-*co*-HV)) with tailoring topography via breath figure technique was presented. The influence of polymer characteristics (molecular weight, copolymer composition) and process parameters (thickness of liquid polymer film, polymer concentration, etc.) on the film structure has been evaluated. The effect of the topography of the obtained films on mesenchymal stem cell growth on films has been examined. These studies will allow consideration of modification of the surface roughness of the implants to influence cellular behavior in vitro.

## 2. Materials and Methods

### 2.1. Polymer Synthesis

The method for obtaining the polymer was previously described [22] and was based on controlled biosynthesis of poly(3-hydroxybutyrate) and its copolymers (Table S1, Supplementary Materials) using a highly efficient producing strain of *Azotobacter chroococcum* 7B (https://www.ncbi.nlm.nih.gov/nuccore/OK077566.1, accessed on 14 September 2021), a non-symbiotic nitrogen-fixing bacterium capable of overproducing PHB. As an additional carbon source for the biosynthesis of the PHB-*co*-HV copolymers, the salt of valeric acid was added into the culture medium in various concentrations. The process of isolating and purifying the polymer from the biomass of the producer strain included: extraction with chloroform, filtration, precipitation with isopropyl alcohol, purification by several cycles of dissolution-precipitation and drying. For the subsequent preparation of films, the polymer was dissolved in chloroform. Physicochemical characterizations of the obtained PHB350, PHB1800, PHB-*co*-HV (9%) and PHB-*co*-HV (17%) are given in Table S1, Supplementary Materials. PHB-*co*-HV 9% and 17% were used in this study as a copolymer with the most sustainable maximum occurrence of valeric acid (17%) and copolymer with half amount of 3-hydroxyvalerate in a polymer chain.

### 2.2. Preparation of Honeycomb-Patterned Films

The silicon substrates were pre-cleaned using «Piranha» solution (H_2_O_2_ (30%)/H_2_SO_4_ = 1:3 (*v*/*v*)) at 120 °C followed by rinsing with distilled water and air dried. PHB and PHB-*co*-HV were dissolved in chloroform at a concentration of 1 to 40 mg/mL. The polymer solution in the volume range of 150 to 450 µL was cast onto the silicon substrate (2 × 2 cm^2^). The solution spread evenly over the substrate, forming a liquid polymer film. This was then followed by humidified air (75% relative humidity, 37 °C) being blown to the deposited polymer solution. The required air humidity was generated by bubbling through a saturated aqueous solution of sodium chloride upon thermostating. The air flow was vertically blown to the surface of the liquid polymer film at a rate of 2.5 L/min from a distance of 1 and 3 cm. The concentration of PHB and PHB-*co*-HV solutions were altered from 1 to 40 mg/mL. After evaporation of chloroform, a thin opaque polymer film remained onto the substrate.

Polymer films with no roughness were prepared as a control without humidified air.

### 2.3. Preparation of Patterned Films on Stamps

Two calibration gratings for an atomic force microscope were used as artificial templates: TGX1 (TipsNano) and TGZ3 (TipsNano). Patterned PHB films were obtained via centrifugation (10,000 rpm, high-speed centrifuge TsV-01/1, Belarus) by applying 30–40 µL of a polymer solution in chloroform with a concentration of 40–100 mg/mL onto the templates. Patterned films were obtained from PHB350 and PHB-*co*-HV(17%) polymers. Forming patterned films on stamps from PHB1800 and PHB-*co*-HV(9%) was found to be impossible due to an inability to adjust the necessary concentrations.

### 2.4. Characterization of Patterned Films

The morphology of the prepared films was examined using a scanning electronic microscope (SEM) equipped with an energy-dispersive X-ray (EDX) spectrometer (JCM-6000 Plus, JEOL, Akishima, Tokyo, Japan). Samples were covered by platinum and SEM images and were made in a high-vacuum mode at an accelerating voltage of 15 kV. The average pore diameter of the patterned films was estimated from SEM micrographs using “JCM-6000 Plus Standard Software ver. 1.6.0”.

The polymer film surfaces were compared based on differences in their pore area and circularity:

Circularity = 4π(area/perimeter^2^), (1)

The equation above describes how regular the pore formed is, ranging from 0 to 1, where a circularity of 0 denotes a formation of an irregularly shaped pore and a value of 1 indicates a formation of a perfectly circular pore.

The samples were selected so that their pores are processable with a sufficient sample size (minimum 13 pores).

ImageJ was used for extracting data from the obtained SEM images using a ‘magic wand tool’ for capturing and outlining the surface pores.

Static water contact angle (θ) of the patterned films was measured by the Sessile drop technique. The images of distilled water droplets (5 µL) on the PHB and PHB-*co*-HV surface were registered using a digital optical camera.

### 2.5. Differential Scanning Calorimetry

The study of the thermophysical characteristics of the obtained patterned films (measurement of melting and crystallization temperatures, melting and crystallization heats, calculation of the degree of crystallinity) was carried out using a DSC 204 F1 Phoenix (Netzsch, Selb, Germany) differential scanning calorimeter. The samples were heated from 25 to 220 °C in an aluminum crucible at a heating rate of 10 K/min in an argon atmosphere. For accurate temperature and enthalpy calibrations in the temperature range from −100 °C to 600 °C, according to the manufacturer’s instructions, a Netzsch calibration kit was used (high purity samples of KNO_3_, In, Bi, Sn, Zn, CsCl, Hg, C_6_H_12_). The crystallinity of the PHB structure (Xc) is calculated as follows (2):

Xc = (ΔHm)/ΔH0m(PHB) × 100%(2)

where ΔHm is the enthalpy change caused by the melting of the test sample, ΔH0m(PHB) is the theoretical value for the thermodynamic enthalpy of melting that would be obtained for 100% crystalline PHB samples (146.6 J/g).

### 2.6. Cells

MSCs (mesenchymal stromal cells) were isolated as previously described [22] from rats‘ bone marrow. Cells were cultivated in a standard α-MEM medium supplemented with 10% FBS and 1% antibiotic (penicillin + streptomycin). Third passage cells were used in the experiment. Samples (1.5 cm^2^ for honeycomb films and 0.5 cm^2^ for stamp films) were sterilized via incubation in ethanol for 1 h. Cells were seeded on samples in 24- or 96-well plates in an amount of 2500 cells per sample.

### 2.7. SEM Imaging

After 2 days of incubation, polymer films with cells were prepared for SEM microscopy according to the standard method. Samples were fixed using fixative solution (2% formalin, 0.2% glutaraldehyde in phosphate buffer solution) and dehydrated with ethanol solutions of different concentrations (30%, 50%, 70%, 82% and 96% ethanol solutions, respectively). HMDS was used as a final fixative solution. Samples were sputtered with platinum. Images were obtained on an SEM microscope TM3000 (Hitachi, Marunouchi, Chiyoda-ku, Tokyo, Japan).

### 2.8. Statistical Analysis

The results were presented as a mean ± standard deviation. The statistical analysis of the obtained data was performed using the Mann–Whitney U test for 2 independent groups analysis and the Kruskal–Wallis one-way ANOVA test for more than 2 independent groups. The value of *p* < 0.05 was considered to be statistically significant.

## 3. Results

### 3.1. Formation of PHB Honeycomb-Patterned Films

**Figure 1:**
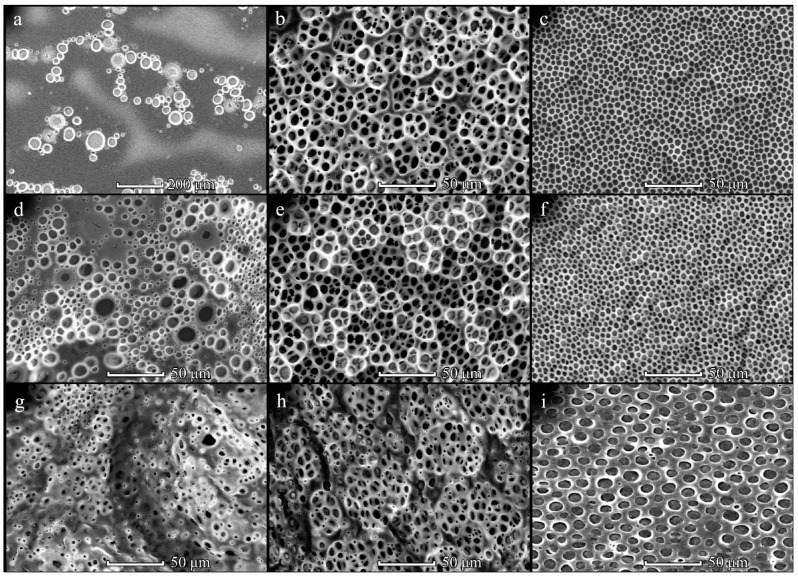
SEM-images of the PHB350 films obtained from solution with concentrations of 5 (**a**,**d**,**g**), 20 (**b**,**e**,**h**) and 40 (**c**,**f**,**i**) mg/mL upon L = 1 cm. The solution volume is 150 (**a**–**c**), 300 (**d**–**f**) and 450 (**g**–**i**) µL.

The surface topography of patterned PHB films was assessed using SEM microscopy. The influence of PHB molecular weight on film topography was examined primarily. It has been determined that low-molecular weight PHB (29 kDa) was not able to form microporous films. Therefore, PHB samples with a higher molecular weight were used for film fabrication. It has been determined that the structure of PHB350 films strongly depends on the polymer concentration (Figure 1). When the PHB350 (Table S1, Supplementary Materials) concentration was less than 20 mg/mL, the disordered morphologies were obtained regardless of the solution volume and distance between the substrate and pipe nozzle (L). The most ordered patterns were fabricated from 40 mg/mL PHB350 solution and L = 1 cm (Figure 1c,f,i). These films had a honeycomb-like structure with one-layered and round-shaped pores, whose size was increased almost 2-fold (from 4.2 ± 0.8 to 8.6 ± 1.7 µm) with enhancement of the casting polymer solution volume from 150 to 450 µL (Table S2, Supplementary Materials). The size of frame elements was also increased under these conditions (Table S2, Supplementary Materials). The tendency to increase in structural elements’ size upon changing L distance from 1 to 3 cm was observed (Table S2, Supplementary Materials). A different film morphology was found for samples obtained from a 20 mg/mL PHB350 solution (Figure 1b,e,h). These samples are 3D microporous structures with several layers of interconnected pores. The average pore size was about 5–6 µm and the frame size was 4–5 µm (Table S2, Supplementary Materials). An increase in the distance L did not have a significant effect on the PHB350 film topography, however, it affected the size of the structural elements (Table S2, Supplementary Materials, Figure 1). The pore size was largely affected by the concentration of the polymers along with its volume, where the distance elevation further enlarged the pores’ sizes.

The PHB350 pore size was elevated along with the concentration and distance increase (Figure S2, Supplementary Materials) with the largest mean area (average pore size) of 194 ± 32.9 μm^2^ (at 3 cm, 20 mg/mL, 450 μL). Further concentration elevation at that point appeared to reduce the average pore size.

The wettability of the patterned films was evaluated via measuring the water contact angle (Table S3, Supplementary Materials). The contact angle of a flat non-pattered PHB350 film was found to be 121.3 ± 3.7°. This value was found to be in a good agreement with the hydrophobic nature of PHB. Compared to the flat surface, patterned PHB350 films displayed a lower contact angle (Table S3, Supplementary Materials). The presence of micro-sized elements on the PHB350 surface resulted in a 1.1 to 1.6-fold decline of the wetting contact angle.

**Figure 2:**
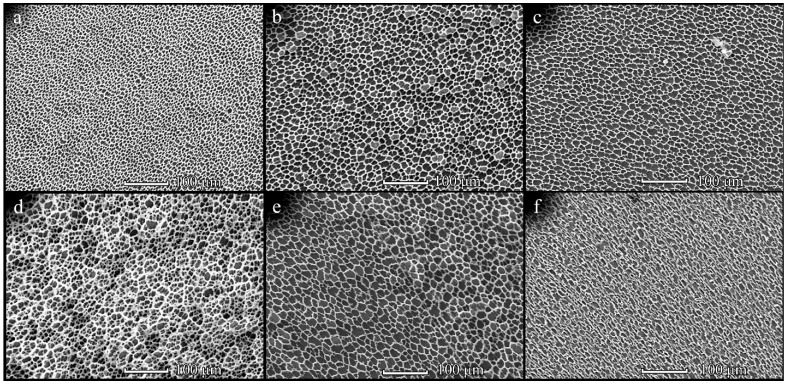
SEM-images of the PHB1800 films obtained from solution with concentrations of 5 (**a**,**d**), 10 (**b**,**e**) and 20 (**c**,**f**) mg/mL upon L = 1 cm. The solution volume is 150 (**a**–**c**) and 450 (**d**–**f**) µL.

**Figure 3:**
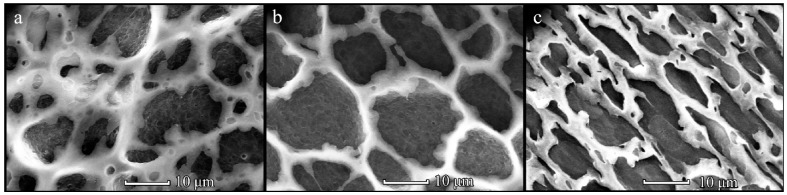
SEM-images of the cells in the PHB1800 films obtained from solution with concentrations of 5 (**a**), 10 (**b**) and 20 (**c**) mg/mL upon L = 1 cm. The solution volume is 450 µL.

An increase in PHB molecular weight from 350 to 1800 kDa led to a significant change in the film topography (Figure 2). As PHB1800 was applied, the formation of mesh-like films with irregularly shaped cells was observed. The size of cells was increased from 5–8 to 7–12 µm upon changing the PHB1800 concentration from 5 to 20 mg/mL, respectively (Figure 2). It should be noted that the topography of the films obtained with the assistance of 150 and 300 µL PHB1800 solutions was identical. However, a further increase in polymer volume led to a change in the structure of the films. For the diluted solution (5 mg/mL), the formation of 3D rather than one-layered structures was observed (Figure 2d). In this case, an increase in the size of the cells with a PHB1800 solution volume enhancement was also observed. The SEM image of the individual cells in high resolution is shown in Figure 3. It can be seen that the aspect ratio (the ratio of the maximum to the minimum size) for elongated cells exceeded the value of 3 and their length reached 25 µm (Figure 3c). The cell size in films obtained from PHB1800 solutions with concentrations of 5–10 mg/mL was 12–20 μm, where the 5 mg/mL sample was characterized by the presence of satellite pores on the polymer frame with a size of 1.5–2 μm (Figure 3a,b).

Increasing the concentration and volume of PHB1800 films significantly increased the pore sizes along with the distance elevation (Figure S3, Supplementary Materials). For comparison, the maximum mean area at a distance of 1 cm was found to be 106 ± 12 μm^2^ (at 1 cm, 20 mg/µL, 300 mL), whereas the maximum mean area at 3 cm was found to be 169 ± 32.7 μm^2^ (at 3 cm, 20 mg/mL, 450 μL).

The presence of a mesh-like structure for PHB1800 films had almost no effect on their wetting (Table S3, Supplementary Materials). The value of the contact angle mainly varied in the range of 117–126 degrees, which correlates with the value for the flat PHB1800 film (118.9 ± 1.6°). Declination of the contact angle (up to 93–106°) was observed for orientated PHB1800 films and samples prepared from the diluted solution (5 mg/mL) with smaller cells (5–8 µm) or 3D structures (Table S3, Supplementary Materials).

### 3.2. Formation of PHB-co-HV Honeycomb-Patterned Films

**Figure 4:**
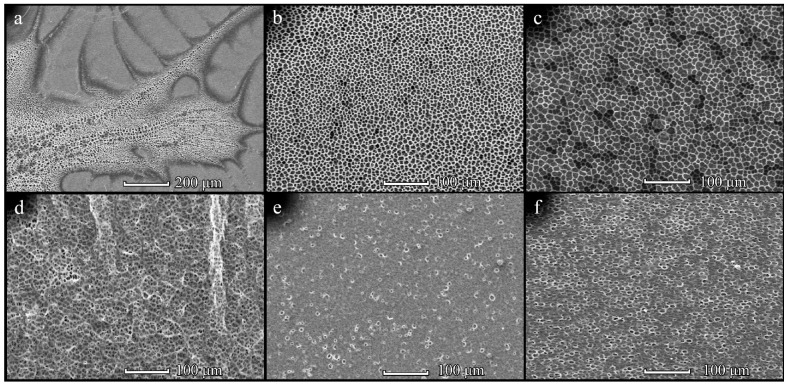
SEM-images of the PHB-*co*-HV(9%) films obtained from solution with concentrations of 10 (**a**,**d**), 20 (**b**,**e**) and 40 (**c**,**f**) mg/mL at L = 1 cm. The solution volume is 150 (**a**–**c**) and 450 (**d**–**f**) µL.

The structure of PHB-*co*-HV(9%) films significantly differed from those obtained from PHB. Among all the studied conditions, the patterned films have been obtained only from 20 and 40 mg/mL PHB-*co*-HV(9%) solutions at the volume of 150 µL (Figure 4). Several layers of interconnected pores with sizes of 3–9 and 5–12 µm were detected for 20 and 40 mg/mL PHB-*co*-HV(9%) samples, respectively. An increase in PHB-*co*-HV(9%) solution volume at these concentrations led to the formation of disordered films with irregularly arranged pores (Figure 4d–f).

Based on the obtained histograms, pore sizes of PHB-*co*-HV(9%) remained unchanged at a distance of 1 cm (Figure S4, Supplementary Materials), however, elevation of the polymer volume at 3 cm from 150 µL to 300 µL resulted in over a 3-fold increase in the average pore size with an average pore area reaching 156.6 ± 28.6 μm^2^ (*p* < 0.05, at 3 cm, 40 mg/mL, 300 μL).

**Figure 5:**
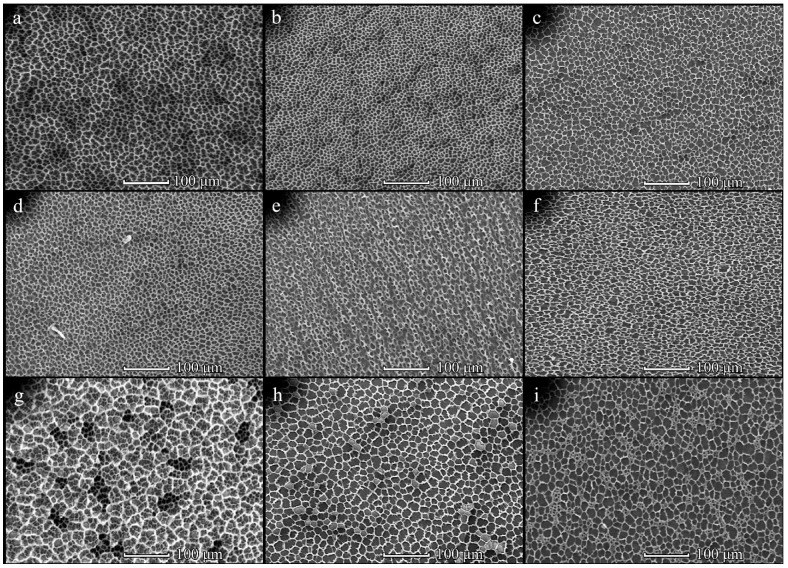
SEM-images of the PHB-*co*-HV(17%) films obtained from solution with concentration of 10 (**a**,**d**,**g**), 20 (**b**,**e**,**h**) and 40 (**c**,**f**,**i**) mg/mL upon L = 1 cm (**a**–**f**) and 3 cm (**g**–**i**). Solution volume is 150 (**a**–**c**) and 450 (**d**–**i**) µL.

An increase in the HV content of the copolymer to 17% significantly changed its ability to form patterned films by a breath figure technique. Under all the studied conditions, the formation of patterned PHB-*co*-HV(17%) films was observed (Figure 5). At the same time, their structure strongly depended on the preparation conditions. The samples obtained using a distance between the substrate and pipe nozzle of 1 cm had a one-layered pore structure with their sizes ranging from 3 to 10 µm (Figure 5a–f).

The PHB-co-HV(17%) polymer film pores were increasing in size along with the concentration and volume elevations for both distances of 1 and 3 cm (Figure S5, Supplemental materials). However, pores at 3 cm demonstrated a larger average size increase with the maximum volume of 262 ± 60.2 μm^2^ (at 3 cm, 40 mg/mL, 450 µL), whereas the largest average pore size at 1 cm was found to be 136 ± 59.4 μm^2^ (at 1 cm, 40 mg/mL, 300 µL).

Increasing the distance L to 3 cm resulted in the formation of structures with interesting topographies. Thus, three-dimensional structures with large pores of 15 µm and subpores of 7 µm can be obtained from the dilute PHB-*co*-HV(17%) solution (10 mg/mL) (Figure 5g). Increasing the concentration to 20 mg/mL led to the formation of single-layer mesh-like films with pore sizes of 13–20 μm, while some of them were covered with a polymer film (Figure 5h). The use of a concentrated PHB-*co*-HV(17%) solution (40 mg/mL) contributed to the formation of a network structure with cell sizes of 14–26 μm, between which the polymer regions of about 20 μm in size with incorporated small (2–3 μm) pores were located (Figure 5i).

It should be noted that fabricated PHB-*co*-HV(9%) films had a higher water contact angle (Table S5, Supplementary Materials) compared to flat film (70.6 ± 2.1°). It should also be noted that the maximal elevation of the contact angle was characteristic for the patterned PHB-*co*-HV(9%) films.

### 3.3. Comparison of Polymer Films

The distribution of films by shape and average area of structural elements shows that varying the conditions makes it possible to obtain films with various characteristics of structural surface elements.

Figure S6, Supplementary Materials, showed a few patterns, where PHB350 pores demonstrated relatively lowest hydrophobicity compared to other polymers. The overall data in Figure S6, Supplementary Materials, suggests that increasing the distance from 1 cm to 3 cm has a positive impact on the polymer hydrophobicity, especially in case of PHB350 and PHB-*co*-HV(17%). The pores of PHB-*co*-HV(17%) appeared to have higher regularity and size than most (but not all) the PHB-*co*-HV(9%) (Figure S7, Supplementary Materials). However, due to a limited availability of processable SEM images of PHB-*co*-HV(17%), the number of PHB-*co*-HV(17%) samples was too small to draw a definite conclusion. In order to confirm the interesting potential differences between PHB-*co*-HV(9%) and PHB-*co*-HV(17%), a study with a larger sample pool is to be conducted in the future.

**Figure 6:**
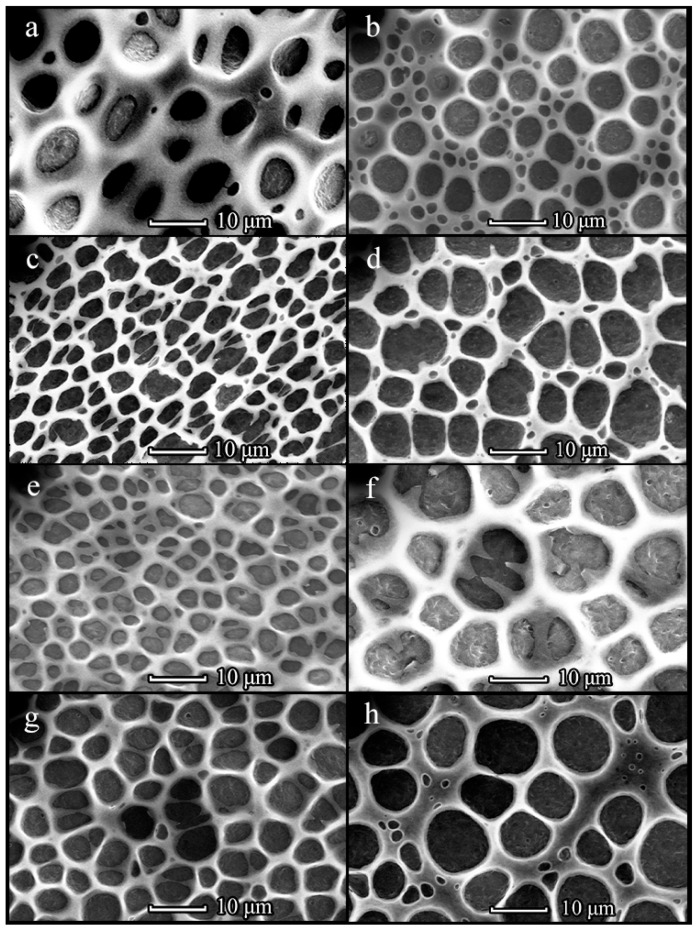
SEM-images of the PHB350 (**a**,**b**), PHB1800 (**c**,**d**), PHB-*co*-HV(9%) (**e**,**f**) and PHB-*co*-HV(17%) (**g**,**h**) films obtained from solution with concentrations of, 20 mg/mL upon L = 1 cm (**a**,**c**,**e**,**g**) and L = 3 (**b**,**d**,**f**,**h**). The solution volume is 150 µL.

Pores on the PHB 350 film surfaces displayed highly regularly shaped and large pores with polymer films at 3 cm, 20 mg/mL, 300 µL and 3 cm, 20 mg/mL (Figure S7, Supplementary Materials), 450 µL having a significant increase in pore sizes whilst retaining the regular shape of the film surface pores. They demonstrated the highest circularity values out of all the studied samples. PHB 1800 surface pore shapes at 3 cm were slightly more regular than the pores at 1 cm, and in case of surface pores at 3 cm, 10 mg/mL, 300 µL and 3 cm, 20 mg/mL, 300 µL formed relatively larger pores in contrast to the polymer films at lower concentrations and at a distance of 1 cm. The pores on PHB1800 were less regular in comparison to the pores on PHB350. No significant patterns were observed in surface pores from PHB-*co*-HV(9%) films. Data from PHB-*co*-HV(17%) demonstrated a significant improvement in pore regularity and size at 3 cm at concentrations and volumes of 20 mg/mL and 150 µL, 20 mg/mL and 300 μL, as well as 40 mg/mL and 300 μL, respectively, when compared to the distance of 1 cm and other concentrations. Pores on PHB-coHV(17%) were more regular than pores on PHB1800. The pores’ regularity was found to be dependent on the conditions for obtaining films. Figure S7, Supplementary Materials, suggests a few potential trends in the studied polymers’ parameters, however, the most evident trend in contrast to others can be observed when the polymers’ distance is changed. This trend can also be observed when comparing the four different polymers at different distances but with the same concentrations and volumes on the SEM images (Figure 6).

Based on data from Figure S8a, Supplementary Materials, the pores produced on the polymer surfaces at a PHB350 concentration of 20 mg/mL revealed the most regular structures (*p* < 0.05) as the film pore size increased, as they demonstrated a higher circularity at higher area parameters compared to other concentrations. Pores produced on the 5 mg/mL polymer surfaces demonstrated the lowest shape regularity (*p* < 0.05). No significant statistical evidence was observed (*p* > 0.05) to suggest important trends in area and contact angle profiles (Figure S8b, Supplementary Materials). Figure S9a, Supplementary Materials, demonstrates a trend, where the distance of 3 cm produced larger pore structures in contrast to 1 cm samples (*p* < 0.05). The distance comparison of the area vs contact angle plot (Figure S9b, Supplementary Materials) demonstrated a similar trend to that observed in Figure S9a, Supplementary Materials. Visual graph analysis suggests that the polymer surface pore films at a distance of 3 cm seemed to have produced relatively larger pores with higher hydrophobicity in contrast to the distance of 1 cm. However, the Mann–Whitney U test (*p* > 0.05) suggests no significant impact of distance on the hydrophobicity and circularity of the polymer surface pores.

The volume comparison in Figure S10a, Supplementary Materials, demonstrated that the pores obtained on polymer surfaces at 450 μL were visually less regular in contrast to the rest. Pores on 150 μL surfaces visually demonstrated high regularity, but produced smaller sized pores when compared to 300 and 450 μL. The volume comparison of the area vs contact angle plot (Figure S10b, Supplementary Materials) demonstrated that 150 µL polymers produced pores with a relatively high and less dispersed hydrophobicity profile in contrast to 300 µL and 450 µL polymers. The polymers at 450 µL showed relatively higher hydrophilicity in contrast to 150 µL and 300 µL. However, the contact angle trend showed no statistical difference between groups, suggesting that there is a small impact of volume change on the hydrophobicity of the polymers. The *p* values of area and circularity were relatively close to 0.05 (0.07 for both), suggesting there is a potential trend to be observed upon adding more repeats to the sample size.

**Figure 7:**
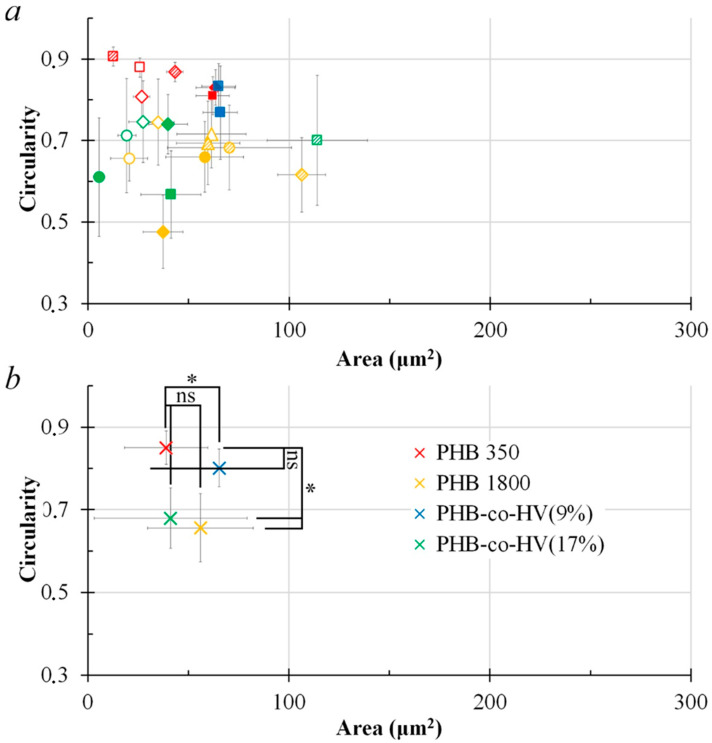
Area vs. circularity plot of structural elements based on their respective concentrations and volumes (150 µL, 300 µL, 450 µL) with the distance L = 1 cm (n = 13). Legends: Polymers: red—PHB 350 kDa; yellow—PHB 1800 kDa; blue—PHB-*co*-HV (9%); green—PHB-*co*-HV(17%). Concentration: ◯—5 mg/mL; △—10 mg/mL; ◇—20 mg/mL; □—40 mg/mL. Volume: blank marker—150 µL; shaded marker—300 µL; filled marker—450 µL (**a**). Average data for each polymer group PHB350, PHB1800, PHB-*co*-HV(9%), PHB-*co*-HV(17%), n = number of data points for each polymer type; * (*p* < 0.05 vs. PHB350) or ns (*p* > 0.05 vs. PHB350) for polymer groups representing area and circularity, the Kruskal–Wallis one-way ANOVA test for more than 2 independent groups (**b**).

**Figure 8:**
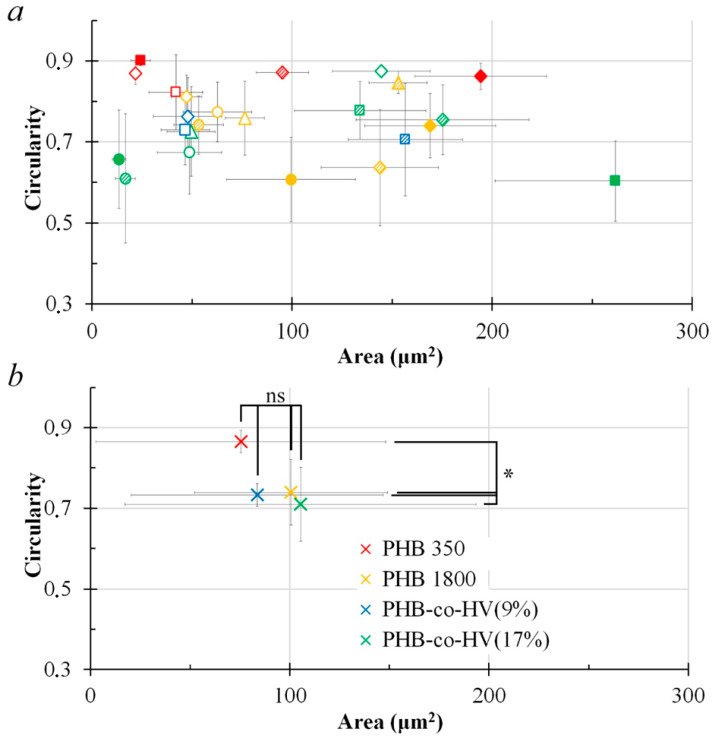
Area vs. circularity plot of structural elements based on their respective concentrations and volumes (150 µL, 300 µL, 450 µL) with the distance L = 3 cm (n = 13). Legends: Polymers: red—PHB 350 kDa; yellow—PHB 1800 kDa; blue—PHB-*co*-HV (9%); green—PHB-*co*-HV(17%). Concentration: ◯—5 mg/mL; △—10 mg/mL; ◇—20 mg/mL; □—40 mg/mL. Volume: blank marker—150 µL; shaded marker—300 µL; filled marker—450 µL (**a**). Average data for each polymer group PHB350, PHB1800, PHB-*co*-HV(9%), PHB-*co*-HV(17%), n = number of data points for each polymer type; * (*p* < 0.05 vs. PHB350) or ns (*p* > 0.05 vs. PHB350) for polymer groups representing area and circularity, the Kruskal–Wallis one-way ANOVA test for more than 2 independent groups (**b**).

Area vs circularity plots in Figure 7 and Figure 8 show that the polymer surface pore structures formed at a distance of 3 cm were more regular and the pore sizes at the same distance were relatively larger (*p* < 0.05) compared to the polymer surface pore at a distance of 1 cm.

**Figure 9:**
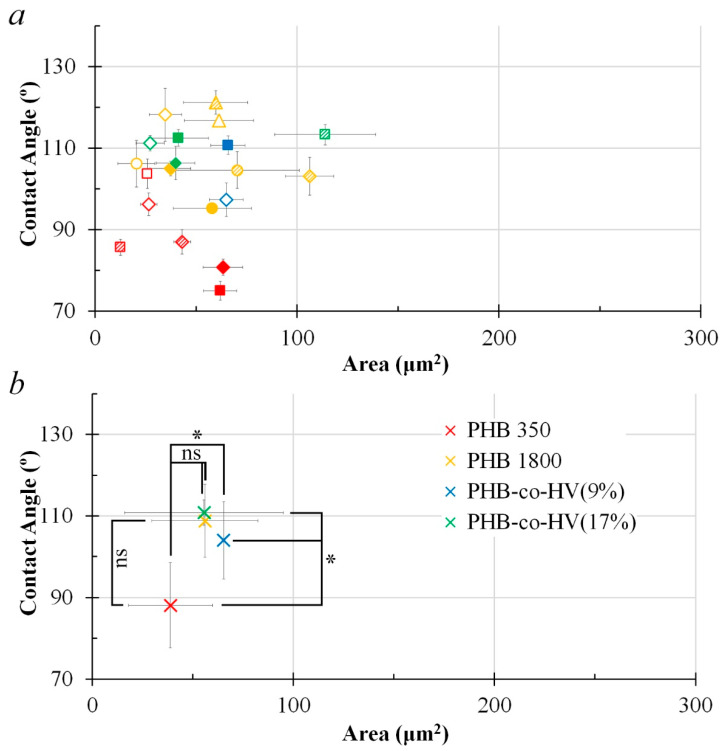
Area vs. Contact Angle plot of structural elements based on their respective concentrations and volumes (150 µL, 300 µL, 450 µL) with the distance L = 1 cm (n = 13). Legends: Polymers: red—PHB 350 kDa; yellow—PHB 1800 kDa; blue—PHB-*co*-HV (9%); green—PHB-*co*-HV(17%). Concentration: ◯—5 mg/mL; △—10 mg/mL; ◇—20 mg/mL; □—40 mg/mL. Volume: blank marker—150 µL; shaded marker—300 µL; filled marker—450 µL (**a**). Average data for each polymer group PHB350, PHB1800, PHB-*co*-HV(9%), PHB-*co*-HV(17%), n = number of data points for each polymer type; * (*p* < 0.05 vs. PHB350) or ns (*p* > 0.05 vs. PHB350) for polymer groups representing area and contact angle, the Kruskal–Wallis one-way ANOVA test for more than 2 independent groups (**b**).

**Figure 10:**
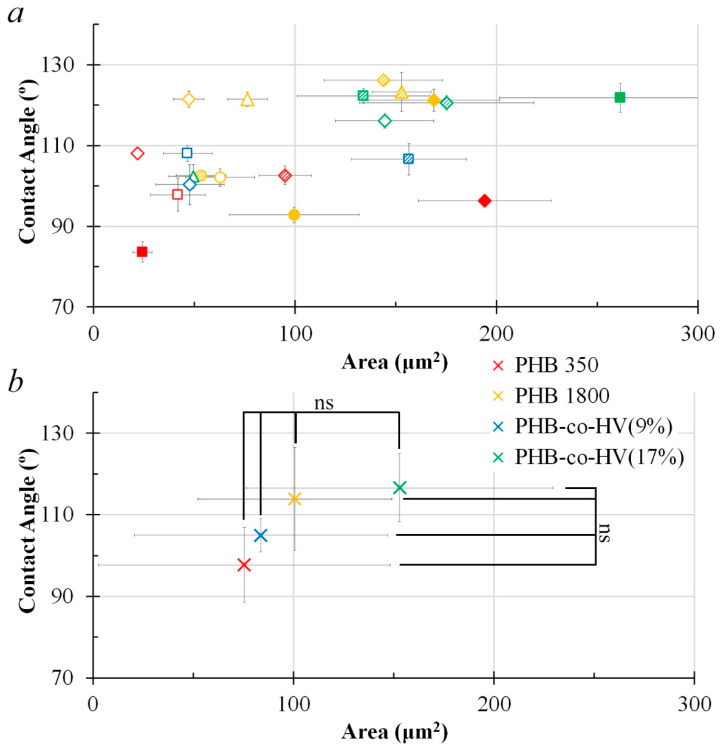
Area vs. Contact Angle plot of structural elements based on their respective concentrations and volumes (150 µL, 300 µL, 450 µL) with the distance L = 3 cm (n = 13). Legends: Polymers: red—PHB 350 kDa; yellow—PHB 1800 kDa; blue—PHB-*co*-HV (9%); green—PHB-*co*-HV(17%). Concentration: ◯—5 mg/mL; △—10 mg/mL; ◇—20 mg/mL; □—40 mg/mL. Volume: blank marker—150 µL; shaded marker—300 µL; filled marker—450 µL (**a**). Average data for each polymer group PHB350, PHB1800, PHB-*co*-HV(9%), PHB-*co*-HV(17%), n = number of data points for each polymer type; ns (*p* > 0.05 vs. PHB350) for polymer groups representing area and contact angle, the Kruskal–Wallis one-way ANOVA test for more than 2 independent groups (**b**).

Data from the averaged area and circularity plots (Figure 9b and Figure 10b) suggests that the most impact caused by changing the distance from 1 to 3 cm was mainly evident in PHB-*co*-HV(9%), where the distance elevation in turn increased the averaged pore circularity (*p* < 0.05). The overall pore size of PHB1800 and PHB-*co*-HV(17%) also significantly improved (*p* < 0.05).

Figure 9b and Figure 10b demonstrate an effect of increasing the distance from 1 to 3 cm on hydrophobicity, where the distance change elevated the PHB350 polymer hydrophobicity when visually assessing the scatter plot. A more thorough study is to be conducted in order to statistically confirm the effect of distance on polymer hydrophobicity in this case (*p* > 0.05).

### 3.4. Cells on Patterned Films Formed on Stamps

**Figure 11:**
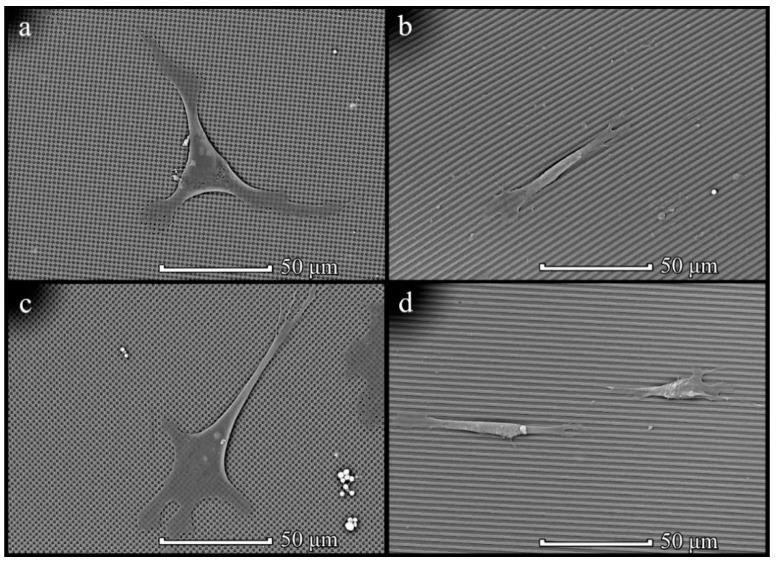
SEM images of MSCs on patterned films prepared on stamps: PHB350 TGX1 (TipsNano) (**a**), PHB350 TGZ3 (TipsNano) (**b**), PHB-*co*-HV(17%) TGX1 (TipsNano) (**c**) and PHB-*co*-HV(17%) TGZ3 (TipsNano) (**d**) polymers.

Assessment of the effect of the patterned film microstructures formed on stamps on cell attachment and growth was studied on two types of microstructures: on TGX1(columns) obtained and TGZ3 (grooves, Tokyo, Japan) obtained films. For the initial assessment of the morphology of MSCs on patterned films formed on stamps, the following samples with cells were examined using SEM after 2 days of incubation: PHB350 TGX1 (TipsNano, Tallinn, Estonia) obtained, PHB350 TGZ3 (TipsNano) obtained, PHB-*co*-HV(17%) TGX1 (TipsNano) obtained and PHB-*co*-HV(17%) TGZ3 (TipsNano) obtained polymer films (Figure 11).

**Figure 12:**
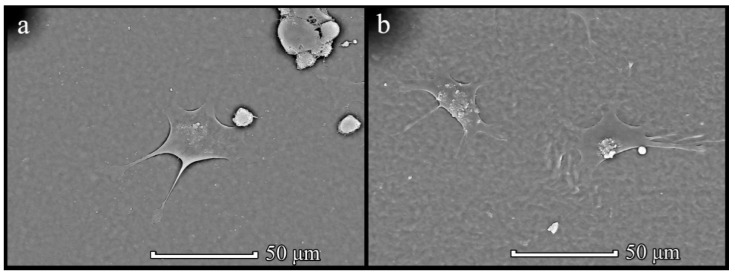
SEM images of MSCs PHB350 (**a**) and PHB-*co*-HV(17%) (**b**) films.

MSCs showed sufficient attachment and growth on all types of films. Furthermore, a tendency in the interactions of MSCs with the structured film surface depending on the microstructure of the surface was found. The cells on the groove/ridge samples showed attachment and further growth along the grooves, and the cells had an elongated shape. Cells on a column-type microstructure attached to the film surface spread out and tended to form point contacts with depressions on the surface. In contrast to the chaotic morphological diversity of cell shapes attached to the surface of PHB350 and PHB-*co*-HV(17%) films without a given microstructure, cells grown on films with a groove and column surface microstructure tended to form certain shapes depending on the microstructure (Figure 12).

### 3.5. Cells on Honeycomb Films

Several types of honeycomb films were selected to compare cells’ attachment to the films’ surface. For the initial assessment of the effect of surface roughness on cells, the following types of polymers were chosen according to differences in structural element forms: PHB350 1 cm 20 mg/mL 150 µL, PHB350 1 cm 40 mg/mL 150 µL, PHB350 1 cm 40 mg/mL 300 µL, PHB1800 1 cm 20 mg/mL 150 µL, PHB1800 1 cm 20 mg/mL 450 µL, PHB1800 3 cm 20 mg/mL 300 µL, PHB-*co*-HV17 3 cm 5 mg/mL 300 µL, PHB-*co*-HV17 3 cm 40 mg/mL 300 µL.

**Figure 13:**
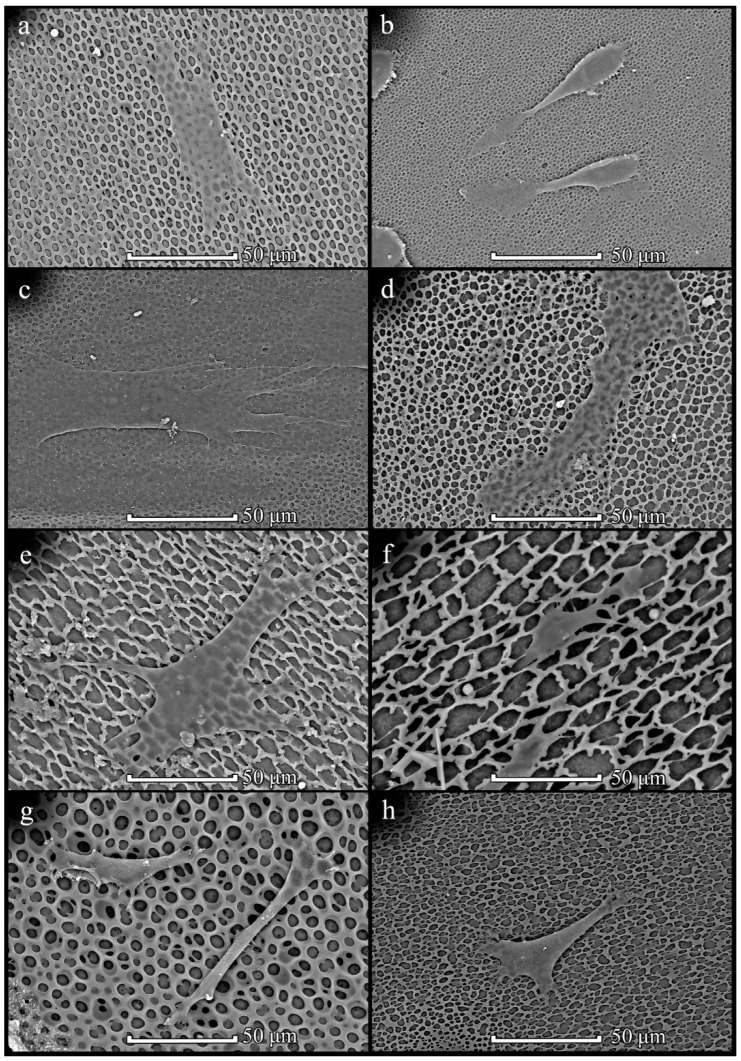
SEM images of MSCs on films: PHB350 1 cm 20 mg/mL 150 µL (**a**), PHB350 1 cm 40 mg/mL 150 µL (**b**), PHB350 1 cm 40 mg/mL 300 µL (**c**), PHB1800 1 cm 20 mg/mL 150 µL (**d**), PHB1800 1 cm 20 mg/mL 450 µL (**e**), PHB1800 3 cm 20 mg/mL 300 µL (**f**), PHB-*co*-HV17 3 cm 5 mg/mL 300 µL (**g**), PHB-*co*-HV17 3 cm 40 mg/mL 300 µL (**h**).

MSCs were seeded on film surfaces in an amount of 2500 cells per sample (1.5 cm^2^) and incubated for 2 days to achieve attachment. SEM images were obtained to compare cell behavior on the surface’s roughness (Figure 13).

Three types of PHB350 were chosen as polymers with the most circular pores and different mean areas, three types of PHB1800 were chosen as irregular pores with different mean areas and two types of PHB-*co*-HV were chosen as films with big regular pores. Thereby the impact of size and circularity of structural elements of the polymer surface on cell attachment could be compared. On films with small round pores (Figure 13a–d), MSCs were flattened by their whole body, whereas on bigger pores (Figure 13e–g), MSCs were more compact and have pseudopodia trying to latch to surface roughness. When concentrated solutions (20 mg/mL) were used, the orientation of the cells was observed; they became elongated along the axis (Figure 13f).

Four types of these polymer films were chosen to demonstrate changes in physical characteristics after their incubation in culture media and with cells growing on them for a week. Table 1 represents the change of main thermophysical characteristics of patterned films after incubation in culture medium with and without cells—melting temperature and degree of crystallinity were calculated based on DSC thermogram data. There is a trend towards an increase in the degree of crystallinity and melting temperature of patterned films after the growth of MSCs on their surface, while incubation of films for the same period of time in a culture medium without cells did not lead to such changes.

This data was in good agreement with our previous studies of the change in the degree of crystallinity during enzymatic degradation and after culturing cells on the surface of standard thick PHB films [23]. This is due to the action of hydrolases and their splitting of the amorphous phase of the polymer, followed by leaching of low molecular weight fractions from it, which are initially present there, which causes such significant changes in the physicochemical properties. Since the hydrolytic degradation of the polymer occurs much more slowly, in the case of incubation simply in a culture medium without the initiation of the process by enzymes, such a noticeable release of oligomers that can change the supramolecular structure of the polymer does not occur.

## 4. Discussion

The effects of concentration, distance and volume on pore size were not fully correlative with relevant existing studies. The effect of distance from the nozzle enhanced the overall polymer pore diameter (Figure S9a, Supplementary Materials), which was the trend found in previous studies on honeycomb polymers [24]. However, the results on the effects of polymer concentration on the pore size were not conclusive, mainly due to the fact that all four polymers were compared at once (Figure S8, Supplementary Materials). The expected trend was to observe a pore size decline upon polymer concentration elevation [18,25]. As to the volume and pore size evaluation, a similar difficulty to the concentration and pore size profile was evident. Nevertheless, it was possible to observe a trend where an increase in volume from 150 to 300 µL resulted in an increased pore diameter (Figure S10a, Supplementary Materials), which correlated with the existing literature [26]. A more thorough and isolated study is required to further assess how each of the parameters affect the pore size and regularity of each polymer, as pore regularity could be a direct result of the polymer preparation stage [27], but it could also be a result of the polymer type, concentration, molecular weight, distance or the casting volume.

The patterned PHB and PHB-*co*-HV films were fabricated by the breath figure method. The key process responsible for the formation of the patterned honeycomb structure by this approach is the condensation of water microdroplets on the polymer surface, which acts as a template for pore formation. Furthermore, these microdroplets are self-organized into a hexagonal array due to Benard–Marangoni convection [18,19]. The size of water droplets and their arrangement determine the structure of the forming polymer film. In turn, the size of microdroplets depends on a number of factors: the relative humidity and speed of the supplied air, its temperature and rate of solvent evaporation from the liquid polymer film [18,28]. Dong and co-authors [18] noted that the growth of water microdroplets on the surface of a liquid polymer film was proportional to the solvent evaporation time, which, in turn, was directly proportional to the volume of the polymer solution applied to the substrate. The regularity of the film structure and pore size is also determined by the ability of the used polymer to concentrate on the interface with the precipitation of a polymer layer around water droplets [16,29]. It can be summarized that the structure of the film fabricated by the breath figure technique was determined by the balance between the evaporation of the solvent, which contributed to surface cooling and altered the viscosity of a polymer layer, the condensation of water microdroplets on the surface of liquid polymer film and their immersion in it. Therefore, the influence of PHB and PHB-*co*-HV concentration, molecular weight, as well as volume of the supplied solution on the film topography were evaluated.

It has been observed that PHB with a molecular weight of 29 kDa failed to form pore structures. It was assumed to happen due to low viscosity of the low-molecular weight PHB solution, resulting in its inability to encapsulate the water microdroplets and prevent their coalescence.

It has been shown that the use of diluted PHB350 solutions (≤5 mg/mL) leads to the formation of disordered films with sparsely located pores. This can be explained by an insufficient amount of PHB350 to form an ordered structure. At the same time, there was a tendency to decrease in pore size whilst increasing the polymer concentration. It can be assumed that at a higher PHB350 content in the solution, it began to precipitate faster on the water microdroplets’ surface, thus enabling their rapid encapsulation in a solid film. Therefore, the water microdroplets did not have time to grow larger, and the resulting pores had small sizes. These results are in a good agreement with literature data. Several authors [18,28,30] have also demonstrated the elevation of honeycomb-like film pore diameters with decreasing polymer solution concentration.

Enhancement of the applied solution volume, as well as the thickness of the liquid polymer film on the substrate resulted in the formation of a three-dimensional structure in the case of a 20 mg/mL PHB350 solution (Figure 1). The formation of such a multilayer structure is explained by an elevation of the solvent evaporation time and the thickness of the formed liquid polymer film, which can be much larger than the diameter of water microdroplets. Apparently, if the time of solvent evaporation and the thickness of the liquid polymer film provide an opportunity for immersing the several layers of water microdroplets into it, then a three-dimensional framework porous structure can be formed after complete evaporation of the solvent. In case of a strongly concentrated solution (40 mg/mL), the large pores were formed due to the high viscosity of the polymer solution in the preforming film.

An increase in the distance from which the humid air was supplied to the liquid polymer film slowed down the solvent evaporation rate and the condensation of water microdroplets. In this case, upon reaching the surface of the liquid polymer film, microdroplets were deposited on the film with a higher polymer concentration. This means that as the distance increases, the effect on the pore size should be similar to the one observed when increasing the PHB350 concentration. In turn, such an effect was observed for a 20 mg/mL PHB350 solution (Table 1). It should be noted that the polydispersity of pores also increased. For the concentrated PHB350 solution (40 mg/mL), the opposite effect was observed. An increase in the distance L resulted in an enhancement of the pore size. This may be explained by the fact that under these conditions, the water microdroplets condense on a film with a high viscosity and can sink into it only when they reach a large size. An increase in the polydispersity of the films was also observed in this case.

It should be noted that all the patterned PHB350 films were characterized by a lower contact angle in comparison to flat samples. Such PHB350 film behavior does not correspond to Wenzel wetting or Cassie–Baxter regimes. It can be assumed that the declination of the contact angle value with the reorientation of PHB350 chains by the interaction of their hydrophilic parts with the water microdroplets upon honeycomb-like structure formation. Probably, the formed porous structure was enriched with polar groups at the pore walls, resulting in lower wetting angles for patterned films compared to the flat PHB350 layer. It should be noted that authors [31] have demonstrated that the fabrication of honeycomb films from NH2-terminated polystyrene results in the enriched content of hydrophilic amino-groups in the cavities.

An approximately 5-fold increase in PHB molecular weight (up to 1800 kDa) resulted in the formation of mesh-like films with large irregular-shaped cells. The polymer molecular weight determines the viscosity of the applied solution. Therefore, PHB1800 formed highly viscous solutions, where the water microdroplets condensed on their surface could not sink into it. Therefore, droplets coalesced onto the polymer surface and could only penetrate it after achieving large sizes. Therefore, in the case of high molecular weight PHB1800, the formation of mesh-like films with a cell size of about 10 μm and an irregular shape was observed. Unlike PHB350, there was an increase in cell size with increasing PHB1800, indicating a different mechanism of film formation for these samples.

Since the formation of patterned polymer films is based on self-organization processes, it was important to establish the effect of the presence of a more hydrophobic unit in the polymer on the overall film topography. Therefore, the possibility of producing patterned films from PHB-*co*-HV with a valerate content of 9 and 17% was studied. It has been determined that only a few conditions make it possible to fabricate patterned films from PHB-*co*-HV(9%). In contrast to PHB-*co*-HV(9%), PHB-*co*-HV(17%) was able to produce patterned films similar to PHB1800. This copolymer predominantly formed films with larger pores (~10–20 µm) due to its high molecular weight.

The visualization of the diversity of cell shapes depending on the morphology of the substrate was the key feature of the experiments in this study. While investigating the relationship between cell shape and the substrate, it was possible to compare the features of MSC attachment to groove-type PHB350 TGZ3 (TipsNano) obtained and PHB-*co*-HV(17%) TGZ3 (TipsNano) obtained polymer films. In both cases, cells were elongated in the longitudinal direction: the cells were elongated along the recesses in the structure, being attached by pseudopodia to neighboring protuberances. Cells attached to the surface of films of the column type had a more spread-out shape than cells on films with no patterns, where they did not have an orientation direction, pseudopodia and were attached to depressions in the microstructure. MSCs on honeycomb patterned films had various shapes, and their elongation and flattening were related to the size of pores. Various studies show that the presence of pores and their sizes have very different effects on different types of cells. In addition, different materials have different effects on cell attachment despite the same pore size and there is no obvious correlation between pore size and cell behavior [32] The size of pores has a significant effect on MSC differentiation and tissue formation. Smaller pores (5 µm) of PLA films, which are similar to PHB by physical characteristics, allows for the formation of thicker tissue during cartilage formation than bigger pores (20 µm). The presence of pores allows cells to be retained on the film and control the area of tissue formation, whereas cells tend to detach from flat film surfaces [33]. In our research, we did not observe the effect of various copolymers on cell behavior, but we can see the effect of pore sizes on cell flattening. Further investigation is required to test the influence of pore sizes of PHB and PHB-*co*-HV films on MSC differentiation.

## 5. Conclusions

SEM is the preferred tool for the characterization of the attachment of cells to films with different surface microstructures. It makes it possible to identify the cell shape using the local features of the substrate topography.

Different film surface topographies obtained via the self-assembly “breath figure” technique had visible effect on the shape of the attached cells. On the surface with smaller pores, the cells were flatter and were adhesive with more surface, whereas on bigger and irregular pores, the cells were tighter, where they scattered pseudopodia in all directions to attach them to roughness. Further investigation is required to evaluate the correlation between cells’ shape changes and their possible differentiation in order to estimate the possibility of controlled differentiation by changing the surface topology. This will allow researchers to use surface patterns of their implants used in surgery to induce cell differentiation into the desired type without additional substances. The impact of low circularity requires further research.

## Figures and Tables

**Table 1:** Thermophysical characteristics of patterned films (1. PHB 350 kDa, 20 mg/mL concentration, 1 cm distance, 150 µL volume; 2. PHB 350 kDa, 40 mg/mL concentration, 1 cm distance, 300 µL volume; 3. PHB 180 kDa, 20 mg/mL concentration, 1 cm distance, 450 µL volume; 4. PHB 1800 kDa, 20 mg/mL concentration, 3 cm distance, 300 µL volume; 5-PHB 350 kDa, 40 mg/mL concentration, 50 µL volume; 6-PHB 350 kDa, 40 mg/mL concentration, 200 µL volume,) fresh, after 1 week of incubation in culture medium and after 1 week of cell growth of MSCs on their surface.

	Initial Films before Incubation/Cell Culture		Films after Incubation in Culture Medium without Cells for 1 Week		Films after 1 Week of Cell Growth on Their Surface	
Polymer Type	Melting Point, °C	Degree of Crystallinity, %	Melting Point, °C	Degree of Crystallinity, %	Melting Point, °C	Degree of Crystallinity, %
**1**	174	61.0	166	62.3	174	66.2
**2**	164	56.0	162	62.3	176	65.8
**3**	166	67.2	176	66.2	180	70.6
**4**	162/170	66.6	178	63.5	180	74.9
**5**	170	63.2	176	63.8	176	68.4
**6**	174	61.0	165	54.9	160	59.4

## Data Availability

Not applicable.

## Supplementary Materials

The following supporting information can be downloaded at: https://www.mdpi.com/article/10.3390/polym14132671/s1, Figure S1: Schematic representation of the data processing workflow; Table S1: Physicochemical properties of PHA films. PHB = poly(3-hydroxybutyrate); PHB-*co*-HV = poly(3-hydroxybutyrate-*co*-3-hydroxyvalerate); Table S2: Size of structural elements in the patterned PHB350 films (µm); Table S3: Water contact angle of the patterned PHB350 films (°); Table S4: Water contact angle of the patterned PHB1800 films (°); Table S5: Water contact angle of the patterned PHB-*co*-HV films (°); Figure S2: Mean area of structural elements of the patterned PHB350 films (°). Data is presented as mean ± standard deviation, *p* < 0.05; Figure S3: Mean area of structural elements of the patterned PHB1800 films (°). Data is presented as mean ± standard deviation, *p* < 0.05; Figure S4: Mean area of structural elements of the patterned PHB-*co*-HV(9%) films (°). Data is presented as mean ± standard deviation, *p* < 0.05; Figure S5: Mean area of structural elements of the patterned PHB-*co*-HV(17%) films (°). Data is presented as mean ± standard deviation, *p* < 0.05; Figure S6: Area vs contact angle plot of structural elements of the patterned PHB350, PHB1800, PHB-*co*-HV(9%) and PHB-*co*-HV(17%) films based on their respective concentrations, volumes (150 µL, 300 µL, 450 µL), and distances (1 cm and 3 cm). Data is presented as mean ± standard deviation; Figure S7: Area vs. Circularity plot of structural elements of the patterned PHB350, PHB1800, PHB-*co*-HV(9%) and PHB-*co*-HV(17%) films. Data is presented as mean ± standard deviation; Figure S8. Mean area vs circularity plot (a) and vs contact angle plot (b) of structural elements based on their respective concentrations (5 mg/mL,10 mg/mL, 20 mg/mL, 40 mg/mL) (a). *p* > 0.05 for groups representing area (a,b) and contact angle (b). *p* < 0.05 for groups representing circularity (a,b); Figure S9. Mean area vs circularity plot (a) and vs contact angle plot (b) of structural elements based on their respective distances (1 cm and 3 cm). *p* > 0.05 for groups representing circularity (a, b) and contact angle (b). *p* < 0.05 for groups representing area (a,b); Figure S10. Mean area vs circularity plot (a) and vs contact angle plot (b) of structural elements based on their respective volumes (150 µL, 300 µL, 450 µL). *p* > 0.05 for groups representing area (a,b), circularity (a,b) and contact angle (b).
