# Supplementary material for: Honeycomb-Structured Porous Films from Poly(3-hydroxybutyrate) and Poly(3-hydroxybutyrate-co-3-hydroxyvalerate): Physicochemical Characterization and Mesenchymal Stem Cells Behavior
Source: Polymers (Basel). 2022 Jun 30;14(13):2671. doi: 10.3390/polym14132671 (PMC9268957; doi:10.3390/polym14132671)
Supplement: Supplementary file 1 [file polymers-14-02671-s001.zip › polymers-1749803-supplementary.pdf]

Supplementary Materials

Honeycomb-structured porous films from poly(3-hydroxybutyrate) and poly(3-hydroxybutyrate-co-3-hydroxyvalerate): physicochemical characterization and stem cells behavior

Viktoryia I. Kulikouskaya, Viktoryia V. Nikalaichuk, Anton P. Bonartsev, Elizaveta A. Akoulina, Nikita V. Belishev, Irina Demianova, Daryana Chesnokova, Tatiana K. Makhina, Garina A. Bonartseva, Kseniya K. Hileuskaya, and Vera V. Voinova

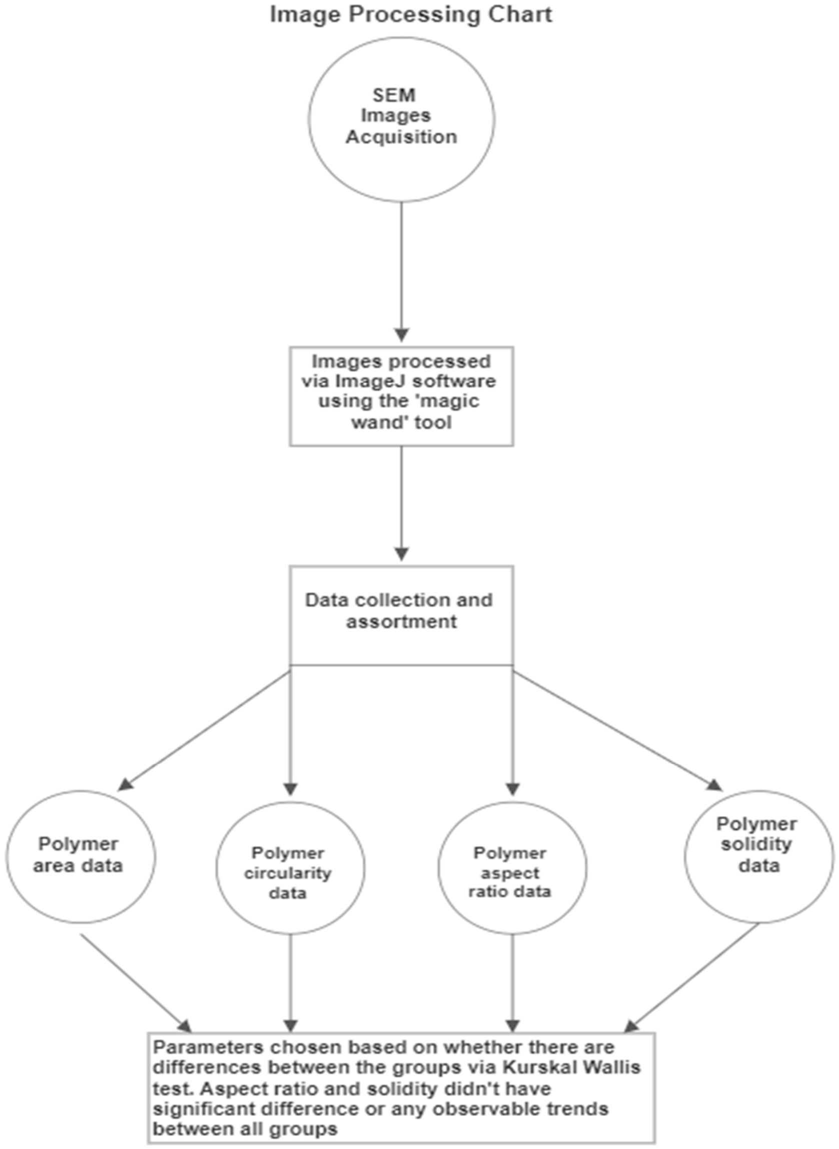

Figure S1. Schematic representation of the data processing workflow.

Table S1. Physicochemical properties of PHA films. *PHB = poly(3-hydroxybutyrate); PHB-co-HV = poly(3-hydroxybutyrate-co-3-hydroxyvalerate).*

**Sample**	**MM, kDa**	**Tc, ℃**	**Tm,℃**	**E, hPA**	**Xc, %**	**Contact angle, º**
**PHB350**	350	87	159/176	2,0±0,02	65	60±2,8
**PHB1800**	1800	80	154/170	2,2±0,06	63	59±3
**PHB-co-HV 9%**	1010	70	155/166	1,0±0,03	44	64±3,6
**PHB-co-HV 17%**	1190	69	154/169	0,4±0,04	34	62±2

Table S2. Size of structural elements in the patterned PHB350 films (μm).

Volume, μL	**PHB350 concentration, mg/mL**							
	**20**				**40**			
	Distance (L), cm							
	1		3		1		3	
	Pore	Frame	Pore	Frame	Pore	Frame	Pore	Frame
150	4.7±1.5	4.4±1.6	3.7±1.8	1.5±0.6	4.2±0.8	2.0±0.9	6.2±2.4	3.2±1.5
300	4.8±0.8	4.6±1.7	4.2±2.8	4.1±1.2	3.7±0.4	1.5±0.4	9.3±1.6	4.9±1.4
450	5.9±1.8	5.3±2.8	5.5±3.9	3.7±2.1	8.6±1.7	5.6±1.6	6.8±2.7	3.8±1.6

Table S3. Water contact angle of the patterned PHB350 films (º).

Volume, μL	**PHB350 concentration, mg/mL**			
	**20**		**40**	
	Distance (L), cm			
	1	3	1	3
150	96.2±2.8	108.1±1.1	103.7±3.6	97.8±4.1
300	87.0±3.0	102.6±2.3	85.7±2.0	104.3±1.9
450	80.8±2.0	96.3±1.0	75.0±2.3	83.6±2.5

Table S4. Water contact angle of the patterned PHB1800 films (º).

Volume, μL	**PHB1800 concentration, mg/mL**					
	**5**		**10**		**20**	
	Distance (L), cm					
	1	3	1	3	1	3
150	106.2±5.7	102.1±2.2	116.8±1.3	121.5±1.8	118.2±6.5	121.5±2.0
300	104.6±4.5	102.6±0.5	121.2±2.9	123.3±4.8	103.1±4.7	126.2±1.1
450	95.2±1.3	92.8±2.0	118.2±1.7	116.9±1.9	105.0±1.8	121.2±2.7

Table S5. Water contact angle of the patterned PHB-*co*-HV films (º).

Volume, μL	Distance (L), cm					
	1	3	1	3	1	3
	PHB-*co*-HV(9%) concentration, mg/mL					
	10		20		40	
150	83.4±2.5	82.2±5.1	97.3±4.1	100.3±5.0	110.7±2.3	108.0±2.0
300	82.0±4.8	85.4±5.3	84.7±3.2	85.6±3.8	102.9±4.9	106.6±3.9
450	89.4±4.3	78.9±2.0	87.0±2.6	82.2±4.2	87.9±6.3	96.0±5.0
	PHB-*co*-HV(17%) concentration, mg/mL					
	10		20		40	
150	106.0±3.3	102.3±3.0	111.2±1.9	116.1±1.8	121.9±0.6	N/A
300	97.5±3.6	96.7±1.9	93.9±3.5	120.6±1.8	113.3±2.5	122.3±1.8
450	87.2±1.0	95.1±2.5	106.3±4.1	121.0±3.9	112.5±2.1	121.6±3.6

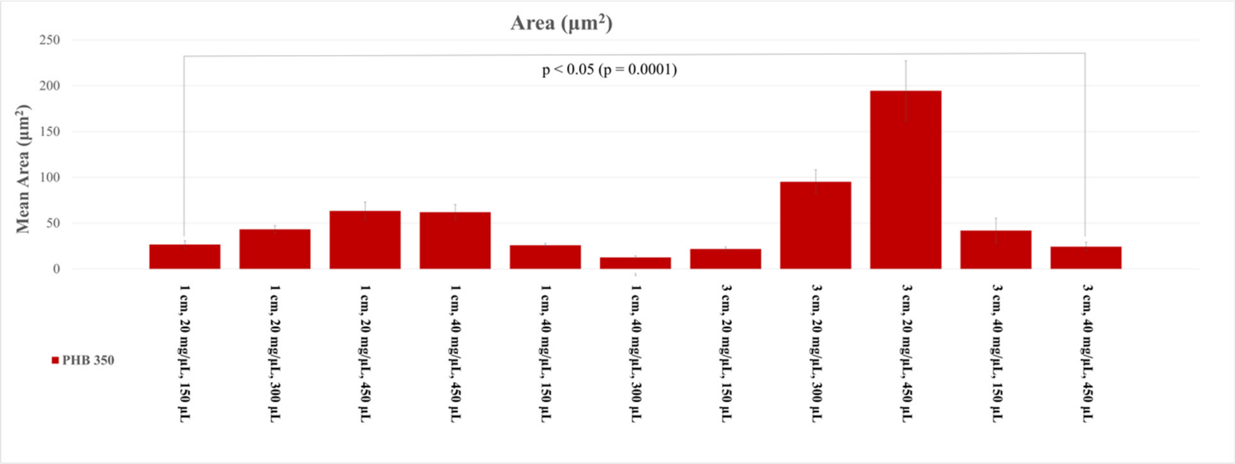

Figure S2. Mean area of structural elements of the patterned PHB350 films (º). Data is presented as mean ± standard deviation, $p < 0.05$.

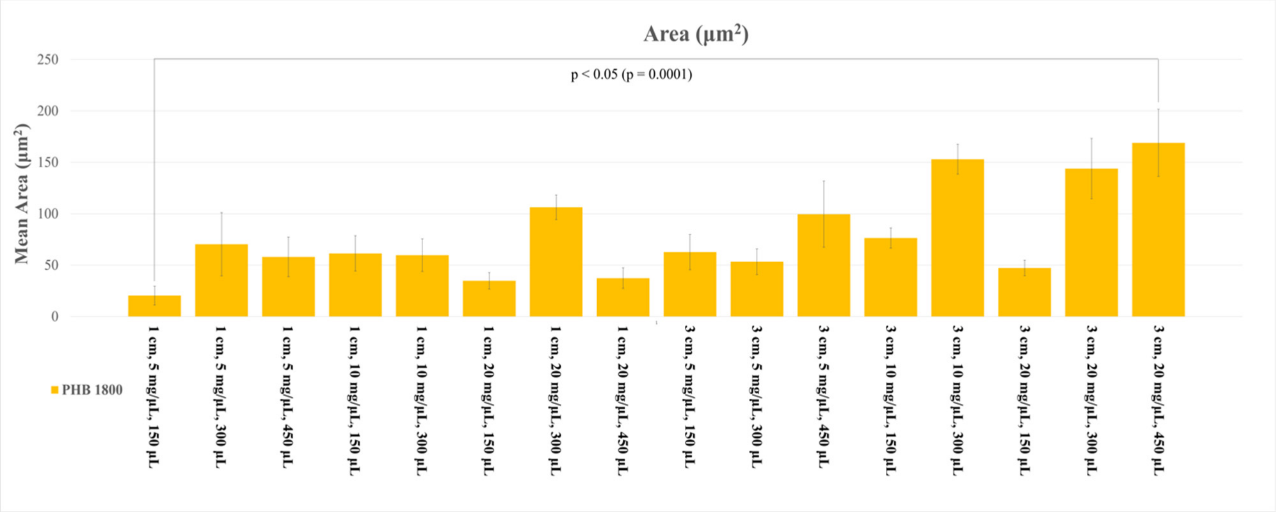

Figure S3. Mean area of structural elements of the patterned PHB1800 films (º). Data is presented as mean ± standard deviation, $p < 0.05$.

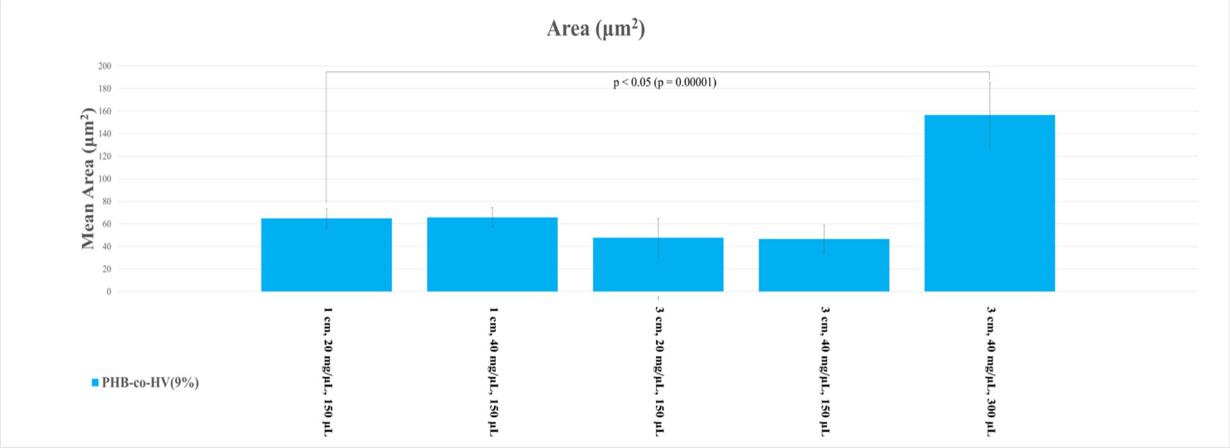

Figure S4. Mean area of structural elements of the patterned PHB-co-HV(9%) films (º). Data is presented as mean ± standard deviation, $p < 0.05$.

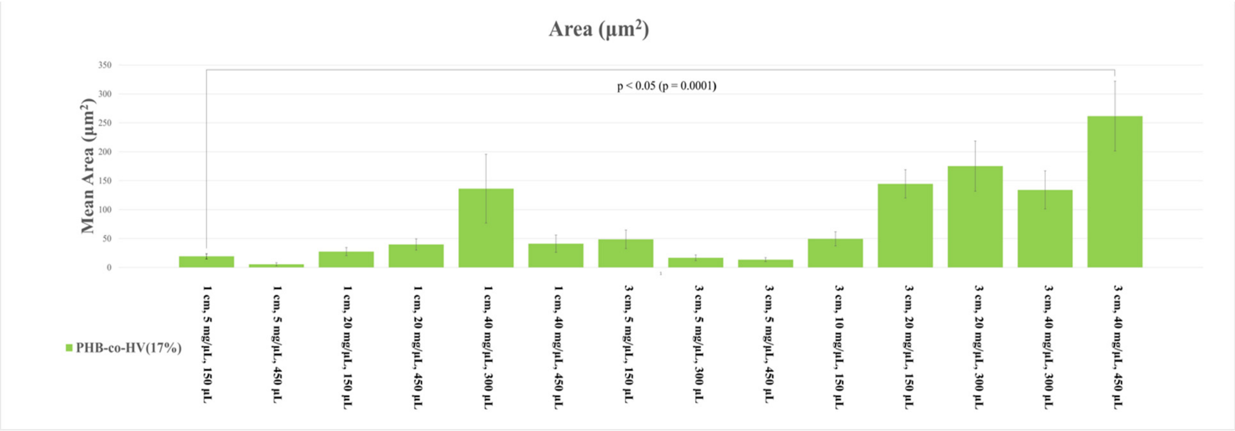

Figure S5. Mean area of structural elements of the patterned PHB-co-HV(17%) films (º). Data is presented as mean ± standard deviation, $p < 0.05$.

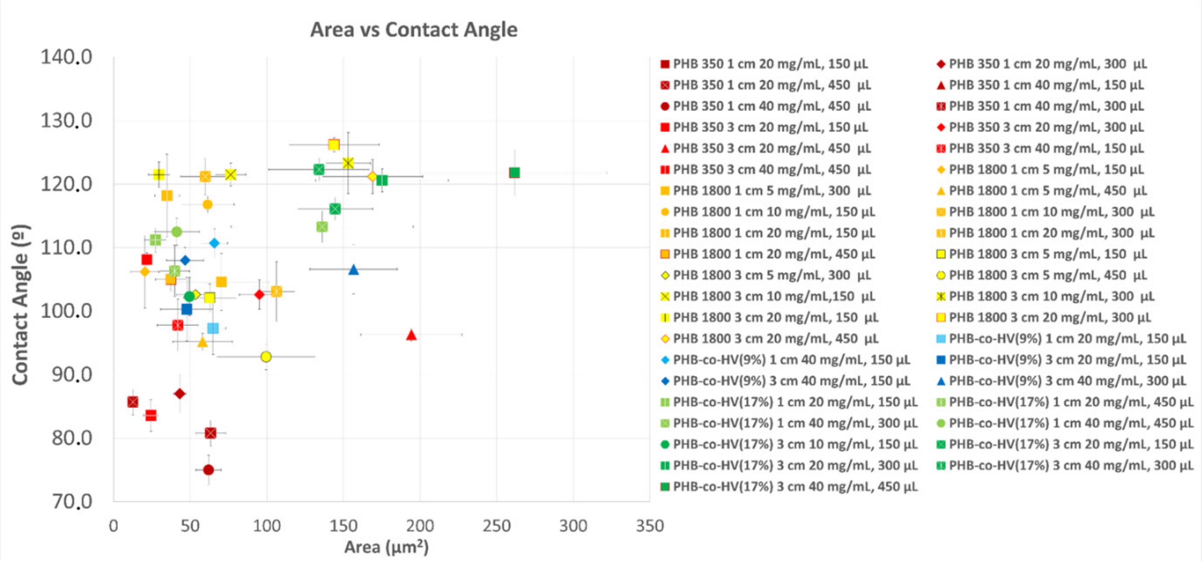

Figure S6. Area vs contact angle plot of structural elements of the patterned PHB350, PHB1800, PHB-co-HV(9%) and PHB-co-HV(17%) films based on their respective concentrations , volumes (150 µL, 300 µL, 450 µL), and distances (1 cm and 3 cm). Data is presented as mean ± standard deviation.

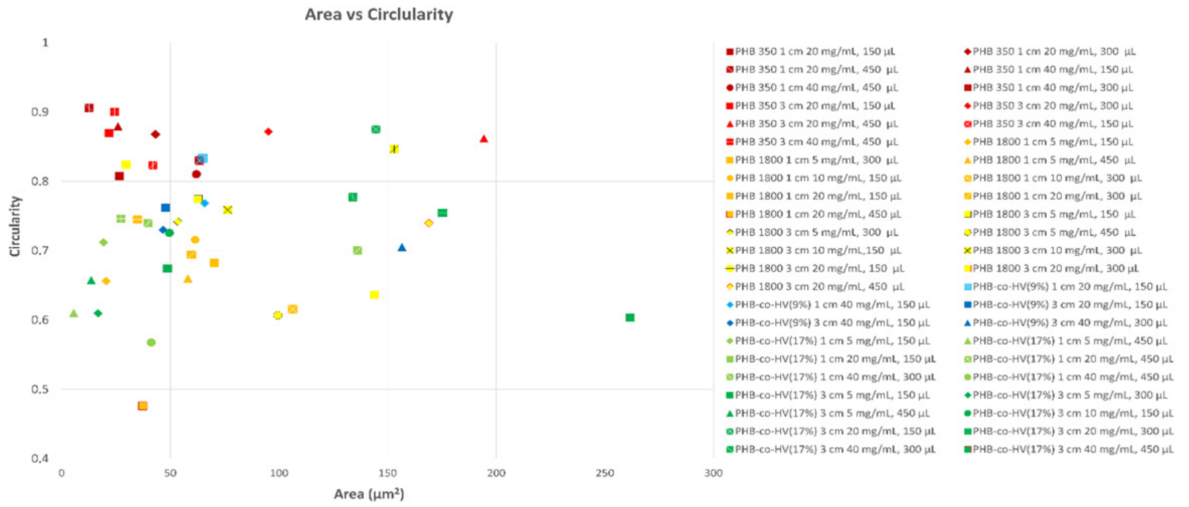

Figure S7. Area vs Circularity plot of structural elements of the patterned PHB350, PHB1800, PHB-co-HV(9%) and PHB-co-HV(17%) films. Data is presented as mean ± standard deviation. $p < 0.05$ for groups representing area and circularity.

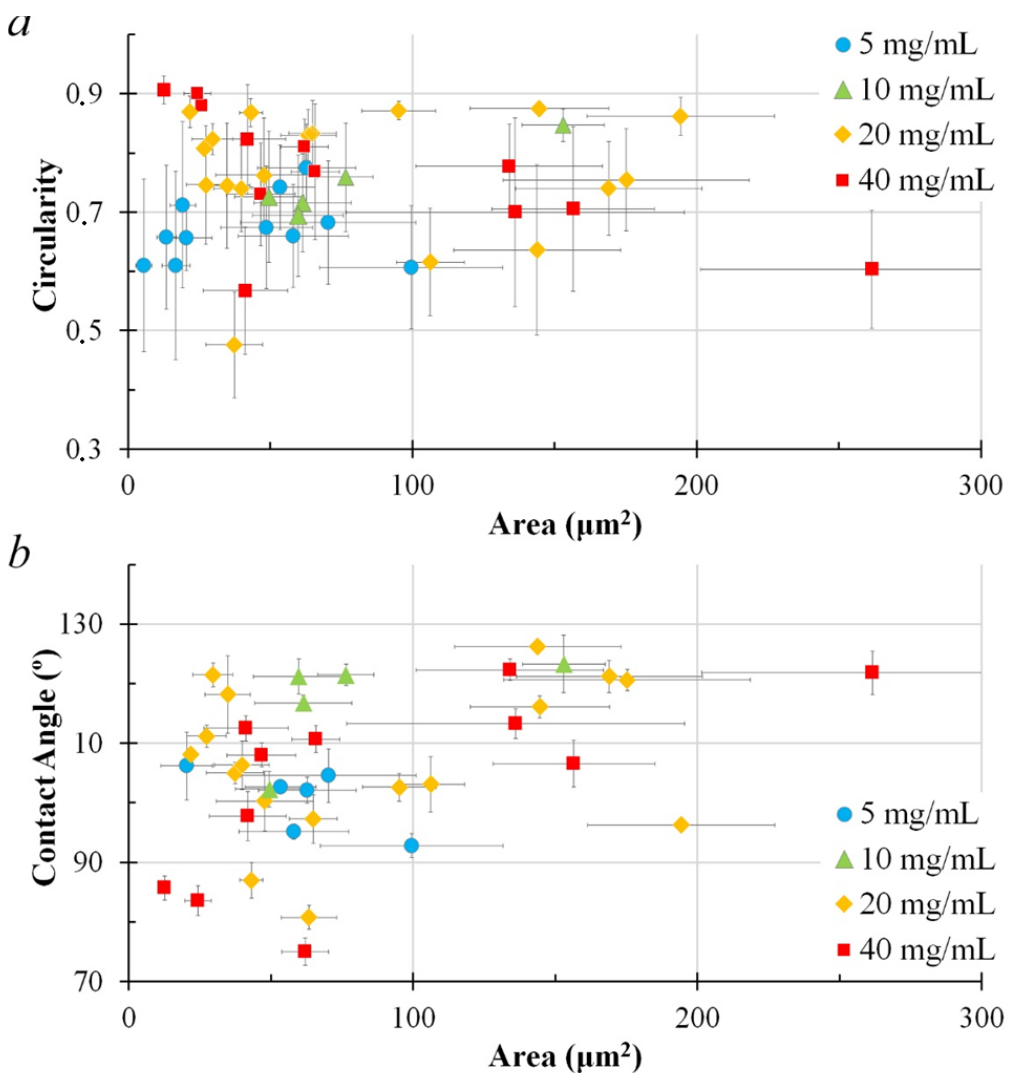

Figure S8. Mean area vs circularity plot (a) and vs contact angle plot (b) of structural elements based on their respective concentrations (5 mg/mL,10 mg/mL, 20 mg/mL,40 mg/mL) (a). $p > 0.05$ for groups representing area (a, b) and contact angle (b). $p < 0.05$ for groups representing circularity, the Kruskal–Wallis one-way ANOVA test for more than 2 independent groups (a, b).

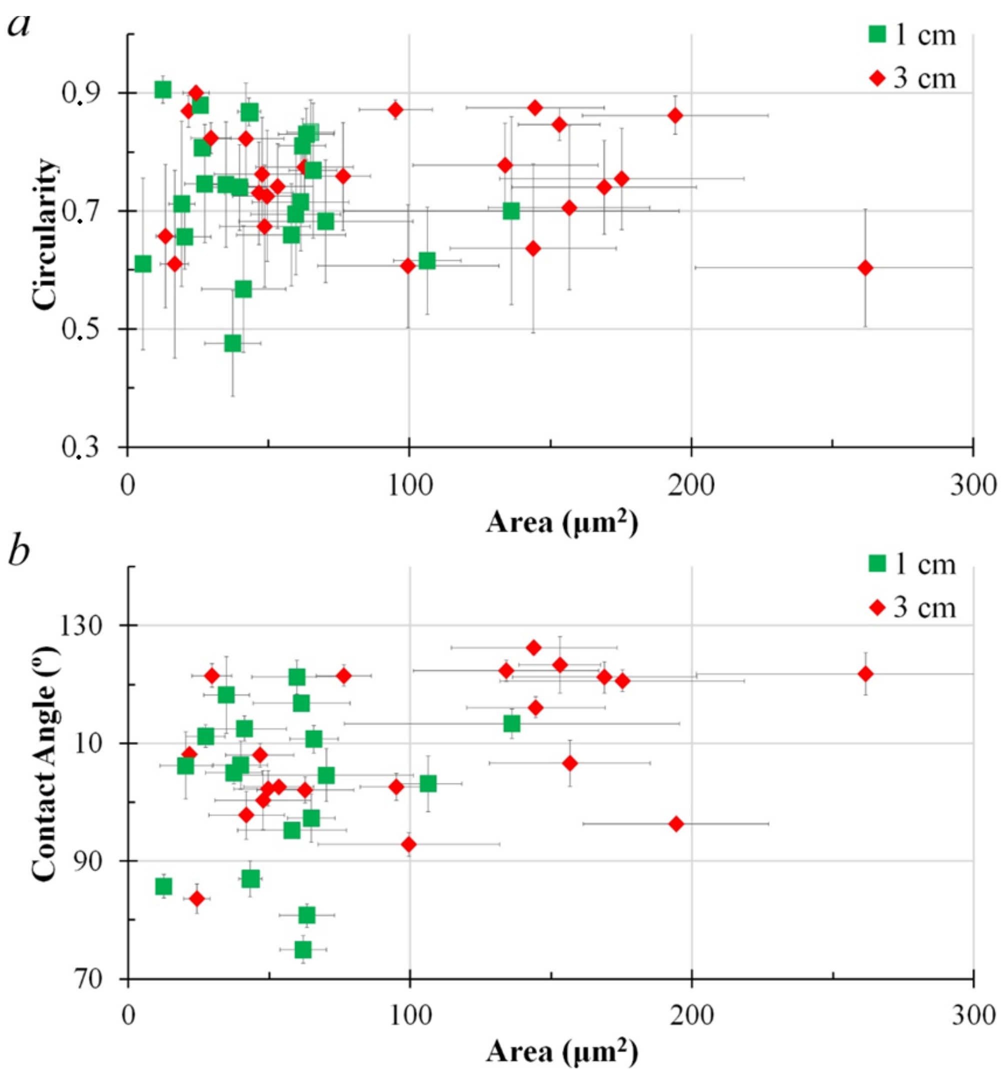

Figure S9. Mean area vs circularity plot (a) and vs contact angle plot (b) of structural elements based on their respective distances (1 cm and 3 cm). $p > 0.05$ for groups representing circularity (a, b) and contact angle (b). $p < 0.05$ for groups representing area, the Mann–Whitney U test for 2 independent groups analysis (a, b).

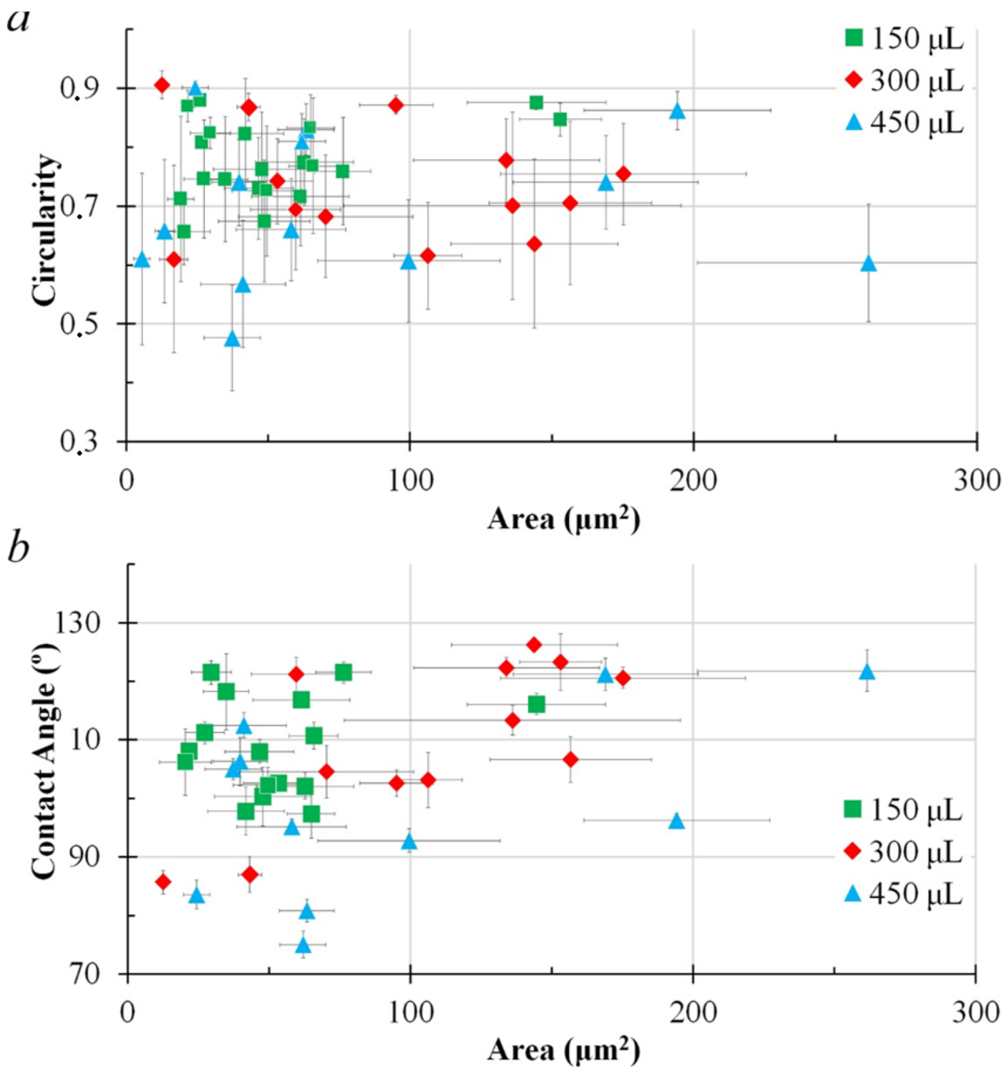

Figure S10. Mean area vs circularity plot (a) and vs contact angle plot (b) of structural elements based on their respective volumes (150 µL, 300 µL, 450 µL). $p > 0.05$ for groups representing area (a, b), circularity (a, b) and contact angle (b), the Kruskal–Wallis one-way ANOVA test for more than 2 independent groups.
